# Enrichment and Correlation Analysis of Serum miRNAs in Comorbidity Between Arnold-Chiari and Tourette Syndrome Contribute to Clarify Their Molecular Bases

**DOI:** 10.3389/fnmol.2020.608355

**Published:** 2021-01-05

**Authors:** Federica Mirabella, Mariangela Gulisano, Mara Capelli, Giovanni Lauretta, Matilde Cirnigliaro, Stefano Palmucci, Michele Stella, Davide Barbagallo, Cinzia Di Pietro, Michele Purrello, Marco Ragusa, Renata Rizzo

**Affiliations:** ^1^Section of Biology and Genetics Giovanni Sichel, Department of Biomedical and Biotechnological Sciences, University of Catania, Catania, Italy; ^2^Section of Child and Adolescent Psychiatry, Department of Clinical and Experimental Medicine, University of Catania, Catania, Italy; ^3^Radiology Unit 1, Department of Medical Surgical Sciences and Advanced Technologies, University Hospital “Policlinico-Vittorio Emanuele”, University of Catania, Catania, Italy; ^4^Oasi Research Institute–IRCCS, Troina, Italy

**Keywords:** circulating microRNAs, liquid biopsies, comorbidity, biological pathways, neuropsychological parameters, neuroimaging parameters

## Abstract

Due to its rarity, coupled to a multifactorial and very heterogeneous nature, the molecular etiology of Arnold-Chiari (AC) syndrome remains almost totally unknown. Its relationship with other neuropsychiatric disorders such as Tourette syndrome (TS) is also undetermined. The rare comorbid status between both disorders (ACTS) complicates the framework of diagnosis and negatively affects the patients' quality of life. In this exploratory study, we aimed to identify serum microRNA expression profiles as molecular fingerprints for AC, TS, and ACTS, by using a high-throughput approach. For this aim, 10 AC patients, 11 ACTS patients, 6 TS patients, and 8 unaffected controls (NC) were recruited. Nine miRNAs resulted significantly differentially expressed (DE): let-7b-5p (upregulated in ACTS vs. TS); miR-21-5p (upregulated in ACTS vs. AC; downregulated in AC vs. TS); miR-23a-3p (upregulated in TS vs. NCs; downregulated in AC vs. TS); miR-25-3p (upregulated in AC vs. TS and NCs; downregulated in ACTS vs. AC); miR-93-5p (upregulated in AC vs. TS); miR-130a-3p (downregulated in ACTS and TS vs. NCs); miR-144-3p (downregulated in ACTS vs. AC; upregulated in AC vs. TS); miR-222-3p (upregulated in ACTS vs. NCs); miR-451a (upregulated in AC vs. TS and NCs; in ACTS vs. NCs). Altered expression of miRNAs was statistically correlated to neuroimaging and neuropsychological anomalies. Furthermore, computational analyses indicated that DE miRNAs are involved in AC and TS pathomechanisms. Finally, we propose the dysregulation of the miRNA set as a potential molecular tool for supporting the current diagnosis of AC, TS, and ACTS by using liquid biopsies, in an unbiased and non-invasive way.

## Introduction

Arnold-Chiari syndrome (AC) is a group of four types of anomalies sharing a malformation of the cerebellum and brainstem. In particular, Chiari malformation type I (CM-I) is characterized by caudal displacement of cerebellar tonsils, which can either be both or singularly herniated, 3 or 5 mm, respectively, below the foramen magnum (FM) (Chiari, 1987; George and Higginbotham, 2011; Urbizu et al., 2013; Markunas et al., 2014).

CM-I is the most common type of anomaly affecting from 1/5,000 to 1/1,000 individuals, affecting females more than males (1.3–1) (Urbizu et al., 2013). Currently, diagnosis is based on symptom evaluation (occipital headaches, ocular disturbances, vertigo, weakness, dysphagia or dysarthria), together with a cranial midsagittal magnetic resonance (MR) analysis (Epstein, 2018). Thanks to the MR analysis, it is possible to detect the degree of cerebellar tonsil herniation not only in patients who show typical debilitating neurologic symptoms, but often also incidentally in those patients who are asymptomatic (Urbizu et al., 2013; Markunas et al., 2014; Epstein, 2018).

Although the etiology of CM-I still remains unclarified and multifactorial, different pathogenic mechanisms have been proposed to define the critical factors causing it (Milhorat et al., 2010; Shoja et al., 2018). Among these, the most accepted consists in a developmental defect of paraxial mesoderm and occipital somites, which leads to a volumetrically-reduced posterior cranial fossa (PCF), a main feature of CMI (Marin-Padilla, 1991; Langridge et al., 2017). The small size of the PCF is unable to hold a normal hindbrain, resulting in tonsillar herniation and an altered hydrodynamic of cerebrospinal fluid (CSF) flow (Barry et al., 1957; Marin-Padilla and Marin-Padilla, 1981; Milhorat et al., 2010; Shaffer et al., 2011).

Furthermore, a genetic basis has been suggested to contribute to CM-I pathogenesis (Speer et al., 2000, 2003). In particular, Urbizu et al. performed a case-control association study on CM-I patients, revealing significant associations with four SNPs within ALDH1A2, CDX1, and FLT1 genes involved in somitogenesis and fetal vascular development (Urbizu et al., 2013).

Several neuropsychiatric disorders may coexist with AC condition (Lacy et al., 2018): anxiety (Caykoylu et al., 2008), epilepsy (Granata and Valentini, 2011), intellectual disability (Grosso et al., 2001), attention deficit hyperactivity disorder (ADHD) and, less recognized, autism spectrum disorders (ASD) (Jayarao et al., 2015; Osuagwu et al., 2016), thus worsening the patients' quality of life (Bakim et al., 2013). Interestingly, AC malformations have been reported to be part of secondary tics and *tourettism* causes (Mejia and Jankovic, 2005).

TS is characterized by multiple involuntary motor and vocal tics, as well as functional and behavioral impairment (Rizzo et al., 2014, 2015; Cirnigliaro et al., 2017; Efron and Dale, 2018). Diagnosis is based on clinical criteria evaluated according to the Diagnostic and Statistical Manual of Mental Disorders (DSM-V), which requires the presence of two or more motor tics and at least one vocal tic, occurring at least once a day and for over 1 year since onset (Ganos and Martino, 2015; Rizzo et al., 2015). TS prevalence is estimated to be ~1% of the population, with a male:female ratio of 4:1. The onset occurs typically in childhood (Robertson, 2015) and in most cases patients improve by late adolescence (Ganos and Martino, 2015).

It has been revealed that up to 85% of children affected by TS present various comorbid neurobehavioural problems (e.g., ADHD, obsessive-compulsive disorder, anxiety, ASD, learning, and sleep disorders), which cause worse functional outcomes (Rizzo et al., 2014; Cirnigliaro et al., 2017; Efron and Dale, 2018). TS pathogenesis is not completely clear, but evidence suggests that complex genetic factors, together with environmental factors, are involved. Susceptibility genes such as *SLITRK1, IMMP2L, CNTNAP2, NLGN4X, LIM* homeobox (*LHX6, LHX8*), *HDC*, and *FLT3* have been identified as associated with TS (Verkerk et al., 2003; Lawson-Yuen et al., 2008; Karagiannidis et al., 2012; Paschou et al., 2012; Baldan et al., 2014; Bertelsen et al., 2014; Yu et al., 2019).

Delineating neuro-psychiatric disorders is still challenging because of the diagnosis complexity and heterogeneity, especially in comorbid conditions. A molecular signature may pave the way to strengthen the traditional approaches for clinical discrimination of TS, AC, and ACTS patients, who show a co-existence of both pathologies.

Over the past few years microRNAs (miRNAs) have attracted increasing attention for several reasons. MiRNAs are small non-coding RNAs, ranging from 18 to 25 nucleotides, which negatively regulate gene expression through post-transcriptional mechanisms. Numerous studies have shown that miRNAs affect cellular pathways and biological processes (Kim and Nam, 2006; Ragusa et al., 2017; O'Brien et al., 2018; Treiber et al., 2019), also playing an essential role in many aspects of neural development (e.g., neurogenesis, neuronal differentiation, maturation, synaptogenesis, neuronal plasticity, neuronal apoptosis) (Bian and Sun, 2011). Furthermore, miRNAs show specific expression and spatiotemporal patterns in mammalian central nervous systems (CNS) (Kosik, 2006; Rao et al., 2013); alterations of miRNA expression have been associated with various brain anomalies and neuropsychiatric disorders (Xu et al., 2010, 2012; Mundalil Vasu et al., 2014; Wu et al., 2015; Hicks et al., 2016, 2019; Alural et al., 2017; Cirnigliaro et al., 2017; Kichukova et al., 2017; Nt et al., 2018; Zadehbagheri et al., 2019), delineating a discriminative molecular signature (Salta and De Strooper, 2012). Furthermore, the evidence that extracellular RNA can be detected in all mammalian body fluids, including peripheral blood (Kosaka et al., 2010; Turchinovich et al., 2011, 2013), opened the way to an important source of biomarkers (Rao et al., 2013; Kichukova et al., 2015; Rizzo et al., 2015): this finding revealed the possibility to analyze the expression of circulating miRNAs (cmiRNAs) through liquid biopsies, in a minimally invasive way (Rao et al., 2013; Nevel et al., 2018). On the other hand, this may objectively support the current diagnosis strategies, unveiling evasive cases that are otherwise not easily detectable.

All these findings underline the importance of using cmiRNAs as a new promising tool in the diagnostic management of Arnold-Chiari Syndrome, Tourette syndrome and their comorbid conditions. To date, only few studies have focused on cmiRNAs in the serum of patients with *pure* TS and in TS comorbid with ASD (Rizzo et al., 2015; Cirnigliaro et al., 2017), while, to our knowledge, no one has characterized cmiRNAs in AC or AC comorbid with TS.

Based on these premises, we exploited a high-throughput approach to investigate cmiRNA expression profiles in sera from AC, TS, and ACTS patients compared to each other and to unaffected controls (NCs), aiming to identify a differential expression among groups. We then evaluated the existence of a molecular relationship between serum miRNA profiles and the scores clinically assigned through a neuropsychological and neuroimaging assessment. Finally, through pathway enrichment analyses, we explored the biological functions of the differentially expressed miRNAs, and thus their potential etiological role in AC, ACTS, and TS pathogenesis.

## Materials and Methods

### Ethics Approval and Consent to Participate

All experiments were approved by the local ethical committee “Comitato Etico Catania 1” (ID: 0024-36-19) prior to sample collection and in accordance with the Helsinki Declaration and its later amendments or comparable ethical standards. Written, informed consent was obtained from parents of all minor age participants (range age 12–13).

### Patient Selection

Twenty-seven Caucasian patients were consecutively recruited from July 2017 to December 2018 at the Section of Child and Adolescent Psychiatry (Department of Clinical and Experimental Medicine, University of Catania).

Patients were affected by AC (*n* = 10), ACTS (*n* = 11), and TS (*n* = 6). They were studied and compared to NCs recruited from local schools (*n* = 8).

### Inclusion Criteria

Patients who were included in the study fulfilled the following criteria for each clinical group:

- AC group: presence of Arnold Chiari malformation type I, defined by a cerebellar tonsillar position (TP) >3–5 mm below the FM; absence of other neurological or metabolic conditions; absence of movement disorders and comorbid conditions such as obsessive-compulsive disorder (OCD) and/or attention deficit/ hyperactivity disorder.
- ACTS group: clinical diagnosis of Tourette Syndrome, according to the criteria of the Diagnostic and Statistical Manual of Mental Disorders, Fifth Edition (DSM-5, APA 2013); the presence of Arnold Chiari type I malformation is based on brain magnetic resonance imaging (MRI): this malformation is defined by cerebellar TP >3–5 mm below the FM.
- TS group: clinical diagnosis of Tourette Syndrome, according to the criteria of the Diagnostic and Statistical Manual of Mental Disorders, Fifth Edition (DSM-5, APA 2013); the absence of other neurological or metabolic conditions such as AC.

Unaffected controls (NCs) were considered neurologically intact children and adolescents, without any history of movement disorder and normal position of cerebellar tonsillar and without chronic neurological, psychiatric, metabolic or genetic diseases. To avoid confounding factors, we did not include either children with minor neuropsychiatric disease (e.g., language delay/disorders or transient tic disorders that did not fulfill criteria for TS) or with minor neurological signs.

### Clinical and Neuroimaging Assessment

A medical history was obtained from all participants and their parents with a focus on neurological and psychiatric conditions. Moreover, all participants underwent a physical and neurological examination and weight, height, head circumference were measured. Venous blood samples were collected. Patients and controls underwent a brain MRI with measurements of posterior fossa (PF) to evaluate the position of cerebellar tonsils.

T1 weighted sagittal brain MRI images were used. All measurements were taken for right and left herniation. Particular attention was paid to the measurements of the following areas: upper posterior cranial fossa, lower posterior cranial fossa, and total posterior cranial fossa; finally, basal, Boogaard, occipital, and tentorial angles were measured (Markunas et al., 2014).

### Neuropsychological Assessment

All participants (AC, ACTS, TS patients and NCs) were assessed by child and adolescent neurologists and psychiatrists (RR; MG) with the following instruments:

- *Wechsler scale* (WISC-III), applied to evaluate Intelligence Quotient (IQ), is a test used to evaluate the intellectual ability of children ranging from 6 to 16 years. To this aim, the assessment provides full-scale intelligence quotient (IQ) scores, as well as Performance IQ and Verbal IQ, two secondary scores (Koyama et al., 2008).
- *The Yale Global Tic Severity Scale* (YGTSS) is an 11-item clinician-rated interview, which is able to evaluate motor and phonic tic severity, considering not only the number and frequency, but also the impairment that tics provoke in the patient. The score of the YGTSS is 0–100, including the impairment section. Higher scores indicate higher severity of symptoms and impairment (Leckman et al., 1989).
- *The Children Yale Brown Obsessive Compulsive Scale* (CY-BOCS) is a semi-structured interview, conducted principally with parents, even if patients are encouraged to participate. This interview is able to assess the severity of obsessive-compulsive symptoms in children. The total score of CY-BOCS ranges between 0 and 40. It is possible to evaluate an obsession and a compulsion score separately. Again, higher scores indicate higher severity of symptoms and impairment (Scahill et al., 1997).
- *The Conners' Comprehensive Behavior Rating Scales* (Conners) is a questionnaire that provides an overview of child and adolescent impairments. Conners is a multi-informant assessment of children and adolescents across multiple settings, with rating forms for parents, teachers, and patients. It is a validated, self- and proxy-rated (parent, teacher) scale used with 12–18-year-olds. It is used to diagnose ADHD and can allow discrimination between subtypes (e.g., predominantly inattentive/ hyperactive-impulsive) (Gianarris et al., 2001).
- *The Child Behavior Checklist* (CBCL) is a report form, which evaluates behavioral problems in children. The CBCL is a validated, parent-rated scale assessing the frequency and intensity of behavioral and emotional difficulties shown by a child over the preceding 6 months. It contains eight syndrome scales (withdrawn, somatic complaints, anxious/depressed, social problems, thought problems, attention problems, delinquent behavior, and aggressive behavior) and two composite scales (externalizing and internalizing problems) (Achenbach and Rescorla, 2001).

### Sample Collection and Processing

Peripheral blood samples from all participants were collected in the morning through a butterfly device, inserted into serum separation collection tubes with Clot activator and gel for serum separation (BD Biosciences). Collection tubes were treated according to current procedures for clinical samples. Tubes were rotated end-over-end at 20°C for 30′ to separate serum from blood cells. Subsequently, they were centrifuged at 3,500 rpm at 4°C for 15′ in a Beckman J-6M/E, supernatants were distributed into 1.5 ml RNase-free tubes, and finally stored at −80°C until analysis (Rizzo et al., 2015).

### RNA Extraction

Extraction of RNA was carried out from 800 μl of serum samples by using a Qiagen miRNeasy Mini Kit (Qiagen, GmbH, Hilden, Germany), according to Qiagen Supplementary Protocol for purification of total RNA (including small RNAs) from serum and plasma (Rizzo et al., 2015). RNA was eluted in 200 μl RNAse-free water and then precipitated by adding 20 μg glycogen, 0.1 volume of 3 M sodium acetate and 2.5 volumes of ice-cold 100% ethanol. After incubation at −80°C overnight, RNA was centrifuged, washed twice in ice-cold 75% ethanol and resuspended in 7 μl RNAse-free water. The yield and quality of the RNA samples were assessed by using NanoDrop Lite Spectrophotometer (Thermo Fisher Scientific, Wilmington, DE, USA).

### MiRNA Profiling

Circulating miRNA expression profiling from serum was performed through the NanoString nCounter system assays by using nCounter Human v3 miRNA Expression Assay Kits (NanoString Technologies, Seattle, USA) and the NanoString platform, according to the manufacturer's instructions. MiRNA profiling was performed on 3 μl (~150 ng) of isolated RNA of 10 AC, 11 ACTS, 6 TS patients, and 8 NCs. RNA samples were processed and immobilized in a sample cartridge for quantification and data collection by using the nCounter Prep Station and Digital Analyzer, respectively. Data analysis was performed using nSolver 3.0 software. Quality of raw data was assessed through an evaluation of imaging, binding density, positive control linearity, and positive control limit of detection parameters. A code-set normalization was applied to minimize technical noise (variations in purification, binding, hybridization efficiency), according to the nSolver analysis software protocol. For each comparison, SAM (Significance of Microarrays Analysis) statistical analyses were computed with MeV (Multi experiment viewer v4.8.1) statistical analysis software. A two-class unpaired test, based on 100 permutations and with a False Discovery Rate (FDR) < 0.05, was computed. We obtained fold change (FC) values, calculating the ratio between the normalized count mean of each group. The data discussed in this publication have been deposited in NCBI's Gene Expression Omnibus (Edgar et al., 2002) and are accessible through GEO Series accession number GSE146509 (https://www.ncbi.nlm.nih.gov/geo/query/acc.cgi?acc=GSE146509).

### Correlation Analysis

In order to investigate whether a linear relationship existed, correlation analyses were computed between the normalized counts of serum miRNAs and the neuropsychological and neurological scores of the study participants for each comparison.

The neuropsychological parameters chosen for this analysis were: total intelligence quotient (TIQ); verbal intelligence quotient (VIQ); performance intelligence quotient (PIQ); Total YGTSS; motor and phonic Yale Global Tic Severity Scale separately; Total CYBOCS; Children's Yale Brown Obsessive Compulsive Scale for obsession and compulsion, separately; Conners scale; Child Behavior Check list internalizing (CBCL-Int); Child Behavior Check list externalizing (CBCL-Ext); total Child Behavior Check list (Total CBCL). Neurological parameters referred to measurements of right and left protrusions (mm), upper posterior cranial fossa area, lower posterior cranial fossa area, total posterior cranial fossa and basal area, Boogaard, occipital, and tentorial angles.

Correlation analyses were performed by GraphPad Prism v8.01 (GraphPad Software, La Jolla, California, USA). Since data were not distributed normally, the Spearman's test was used, applying two-sided *p*-values. Statistical significance was established at *p* ≤ 0.05.

### Computational Analysis

To investigate the biological functions of all the DE miRNAs and their potential etiological involvement in AC, ACTS, and TS, the DIANA-mirPath v.3 web server (Vlachos et al., 2015) and miRNet (Fan et al., 2016) tool were used for functional enrichment analyses from KEGG (Kyoto Encyclopedia of Genes and Genomes) and Reactome gene annotation databases. A class-specific functional enrichment analysis was performed to separately consider miRNAs that were found DE for each pathological condition with respect to the NC group. Fisher's exact *t*-test with FDR correction and Hypergeometric test (*p* ≤ 0.05) were used for enrichment analysis.

## Results

### Demographics

We recruited a total of 35 children from various socio-economic contexts; 10 affected by Arnold Chiari Syndrome (AC) (M:F = 6:4), mean age 13.1 (±3.1) (age range 9–17); 11 affected by Arnold Chiari Syndrome and comorbid Tourette Syndrome (ACTS) (M:F = 9:2), mean age 12.3 (±2.7) (age range 8–16 years); 6 affected by Tourette Syndrome (TS) (M:F = 6:0), mean age 13.8 (±2) (age range 11–16); 8 unaffected controls (NCs) (M:F = 8:0), mean age 12.6 (±2.6) (age range 9–16 years), recruited from local schools. Demographic and neuropsychological characteristics of the clinical sample are shown in Table 1.

**Table 1:** Demographic and neuropsychological characteristics of the clinical sample.

	**ACTS**	**AC**	**TS**	**NC**
Number of participants (36)	11	10	6	8
Sex (M:F)	9:2	6:4	6:0	7:1
Mean age (years)	12.3 (±2.7)	13.1 (±3.1)	13.8 (±2)	12.6 (±2.6)
**IQ**				
TIQ	79.8 (±23.3)	74.3 (±16.3)	96.3 (±11.2)	88.6 (±9.4)
VIQ	79.9 (±22.5)	74.9 (±17.2)	95.3 (±11.3)	86.2 (±9.3)
PIQ	86.7 (±21.6)	77.8 (±17.4)	98.6 (±16.4)	93.1 (±8.5)
**YGTSS**				
Total	19.9 (±8.3)	0	22.5 (±7.2)	0
Motor	12 (±4.1)	0	13.6 (±4.7)	0
Fonic	7.9 (±5.4)	0	8.8 (±2.9)	0
**CYBOCS**				
Total	14.4 (±5.4)	8.2 (±2.8)	18 (±9.7)	7.2 (±3.1)
Obsessions	6.5 (±2.2)	4 (±2.2)	9.3 (±4.8)	4 (±2.7)
Compulsions	7.9 (±3.4)	4.2 (±1.2)	8.6 (±5.3)	6.6 (±9.5)
**CBCL**				
Total	54.3 (±34.4)	26.8 (±12.1)	48.1 (±15.3)	24.9 (±5.8)
Internalizing	16.6 (±11.3)	8.7 (±3.7)	11 (±3.4)	9 (±4.9)
Externalizing	21.4 (±11.9)	10.9 (±6.2)	18.5 (±6.6)	6.4 (±2.6)
Conners	29.5 (±22.3)	25.1 (±14.1)	48.1 (±15.3)	11.2 (±4.5)

*Standard deviation is shown between parenthesis. ACTS, Arnold Chiari + Tourette Syndrome; AC, Arnold Chiari; TS, Tourette Syndrome; NC, Normal Control; TIQ, Total intelligence quotient; VIQ, Verbal intelligence quotient, PIQ: performance intelligence quotient; YGTSS, Yale Global Tic Severity Scale; CYBOCS Tot, Children Yale Brown Obsessive Compulsive Scale total score; CBCL-Tot, Child Behavior Check List*.

*According to two-tailed Mann–Whitney U test, there were no statistically significant sex differences between groups in the comparisons between TS and AC (p-value = 0.2335), TS and ACTS (p-value = 0.2952), TS and NC (p-value > 0.999), NC and AC (p-value = 0.313), NC and ACTS (p-value >0.999), AC and ACTS (p-value = 0.292) groups*.

### Neuropsychological Findings

- Intelligent quotient: with regards to the intelligence quotients (Total, Verbal, and Performance), measured with Wechsler scales, the TS group showed the highest mean value; no statistically significant differences were found in the comparison between clinical groups, except AC vs. TS (*p*-value = 0.005).
- Tourette Syndrome: with regards to tic evaluation, performed with YGTSS, we found scores equal to zero in the AC group and in the normal control group: this could be explained by the exclusion of patients with a tic who did not fulfill criteria for TS to avoid confounding factors (see section Inclusion). TS presented higher scores compared to ACTS, but these differences were not statistically significant in any subscale (motor, vocal, total).
- Obsessive compulsive disorder: with regards to the evaluation of obsessions and compulsions, performed by CYBOCS, AC, and NC presented statistically significant lower scores compared to TS and ACTS. These scores are not able to identify, by themselves, an obsessive-compulsive disorder, but could be the expression of obsessive and/or compulsive behavior that does not interfere with the normal life of the patient. Comparing TS and ACTS groups, TS presented the higher scores, and these scores are identifiable with OCD disorder. No statistically significant differences were found, even if the disorder in ACTS is less severe than in TS.
- Behavioral Problems: with regards to behavioral problems measured with CBCL and Conners' scales, we found the following results: ACTS presented higher scores (total, internalizing, and externalizing), but no statistically significant differences were found with the TS score group. The scores presented in the AC and NC groups were statistically significantly lower compared to the other clinical groups.

Finally, with regards to the Conners' score, the TS group presented the highest statistically significant score compared with the other clinical groups and the NC group. In the comparison between AC and ACTS, no statistically significant differences were found; on the other hand, the score of the NC group was statistically significantly lower compared to the three clinical groups.

### Neuroimaging Measurements

Patients and unaffected controls underwent MRI and all the measurements described in methods section were performed. All measurement details are reported in Table 2. By applying 2-tailed Student *t* tests, statistically significant differences were found between the areas of total PCF between ACTS and NC (*p*-value = 0.0053); AC and TS (*p*-value = 0.0316); AC and NC (*p*-value = 0.0053) groups. No statistically significant differences were found in all the other comparisons.

**Table 2:** Neuroimaging measurements.

	**ACTS**	**AC**	**TS**	**NC**
Protrusion right tonsilla (mm)	4.8 (±1.3)	5.9 (±2)	not applicable	not applicable
Protrusion left tonsilla (mm)	4.5 (±2.6.)	5.8 (±2.5)	not applicable	not applicable
Upper posterior cranial fossa area	1129.6 (±193.4)	1099.2 (±128.9)	1197.2 (±231.6)	1142.9 (±159.9)
Lower posterior cranial fossa area	1971.5 (±305)	1717.7 (±557.8)	1982.6 (±257)	1997.8 (±431.4)
Total posterior cranial fossa area	3101.1 (±323.3)	2738.7 (±487.7)	3265.6 (±288.7)	3362.7 (±274.2)
Basal angle	135.2 (±4.1)	136.4 (±2.7)	131.7 (±6)	131.7 (±6)
Boogaard angle	118.7 (±7.4)	124.7 (±6.3)	120.3 (±,7)	118.1 (±4.7)
Occipital angle	137.5 (±7.2)	132.3 (±7.4)	137.1 (±8.8)	137.2 (±7.8)
Tentorial angle	77.7 (±6.8)	79.8 (±10.8)	82.5 (±8.2)	83.9 (±8.4)

*Standard deviation is shown between parenthesis. ACTS, Arnold Chiari + Tourette Syndrome; AC, Arnold Chiari*.

### Circulating miRNA Expression Profiling

A high-throughput expression analysis of 800 microRNAs in sera of 10 AC, 11 ACTS, 6 TS patients, and 8 NC subjects was computed by using nCounter NanoString technology.

**Figure 1:**
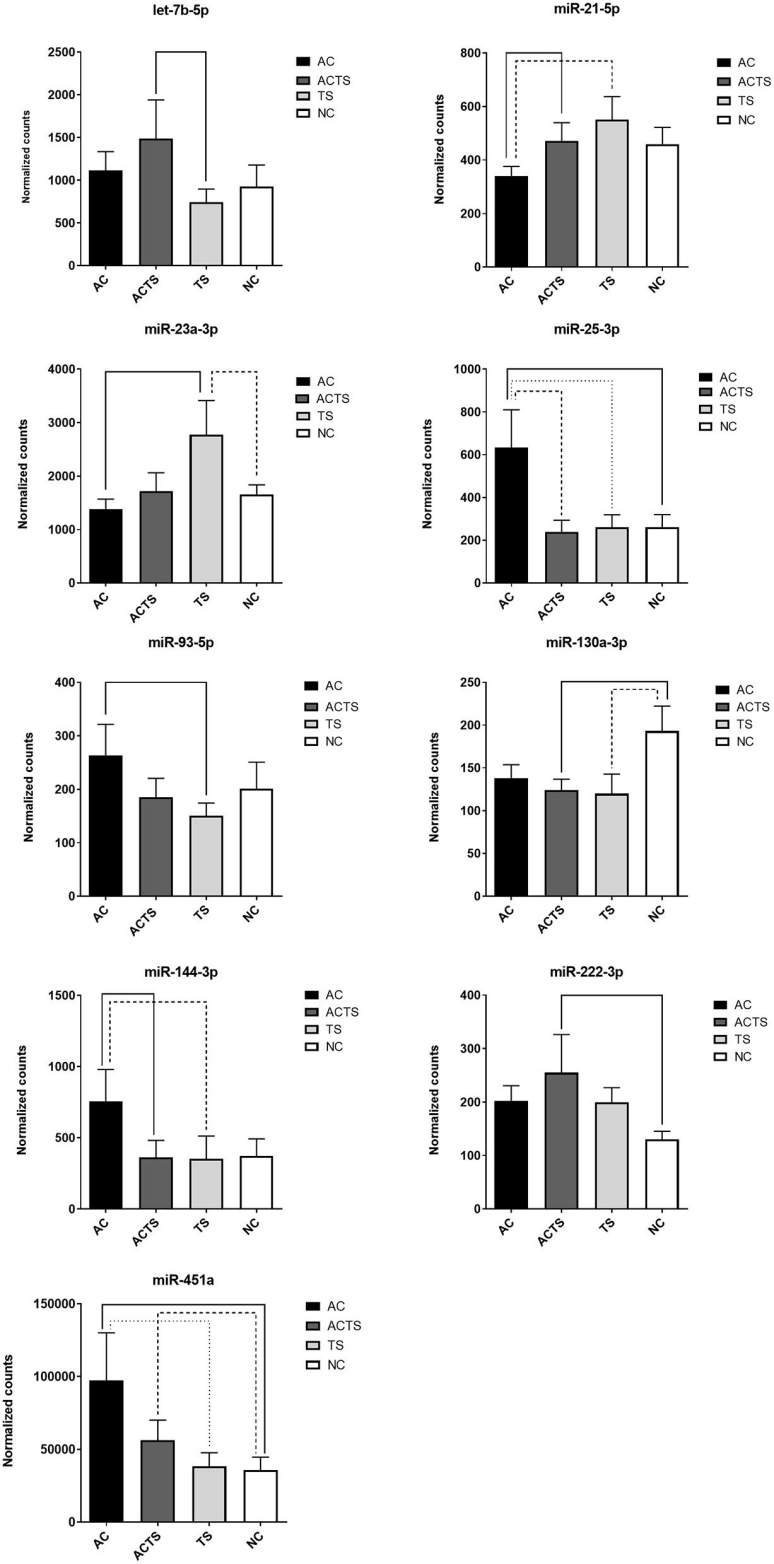
DE-miRNA expression in the serum of AC, TS, ACTS, and NC subjects. Bar plots of the relative expression of the 9 miRNAs for each pairwise comparison: let-7b-5p, miR-21-5p, miR-23a-3p, miR-25-3p, miR-93-5p, miR-130a-3p, miR-144-3p, miR-222-3p, and miR-451a. Each pair whose miRNA dysregulation was found statistically significant (FDR< 0.05) is linked by a continuous or dotted line. Data are shown as means with the standard error of the mean (SEM). Y-axis represents the means of normalized counts with standard error.

We identified 9 miRNAs as significantly differentially expressed in sera from the different groups (Table 3). More specifically: (1) let-7b-5p upregulated in ACTS compared to TS patients; (2) miR-21-5p upregulated in ACTS compared to AC patients and was downregulated in AC compared to TS patients; (3) miR-23a-3p was upregulated in TS compared to NCs and was downregulated in AC compared to TS patients; (4) miR-25-3p was upregulated in AC compared to TS patients and NCs and was downregulated in ACTS compared to AC patients; (5) miR-93-5p was upregulated in AC compared to TS patients; (6) miR-130a-3p was downregulated in ACTS and TS patients compared to NCs; (7) miR-144-3p was downregulated in ACTS compared to AC patients and upregulated in AC compared to TS patients; (8) miR-222-3p was upregulated in ACTS compared to NCs; (9) miR-451a was upregulated in AC compared to TS patients and NCs, and in ACTS patients compared to NCs, with a FDR < 0.05 for each pairwise comparison. The relative expression of DE miRNAs is shown in Figure 1.

**Table 3:** Dysregulation of 9 miRNAs in different pairwise comparisons.

**DE miRNA**	**AC Vs. NC**	**ACTS vs. NC**	**ACTS vs. AC**	**TS vs. NC**	**AC vs. TS**	**ACTS vs. TS**
let-7b-5p	-	-	-	-	-	2
miR-21-5p	-	-	1.38	-	−1.62	-
miR-23a-3p	-	-	-	1.67	−2	-
miR-25-3p	2.43	-	−2.65	-	2.44	-
miR-93-5p	-	-	-	-	1.75	-
miR-130a-3p	-	**–**1.56	-	**–**1.61	-	-
miR-144-3p	-	-	−2.09	-	2.15	-
miR-222-3p	-	1.95	-	-	-	-
miR-451a	2.73	1.58	-	-	2.53	-

*Expression FC values of each DE miRNA are reported for different comparisons, only when statistically significant (FDR < 0.05); -, value not reported because FDR > 0.05*.

### Correlation Analysis Between Serum miRNA Expression Levels and Neuropsychological, Neurological Parameters

**Figure 2:**
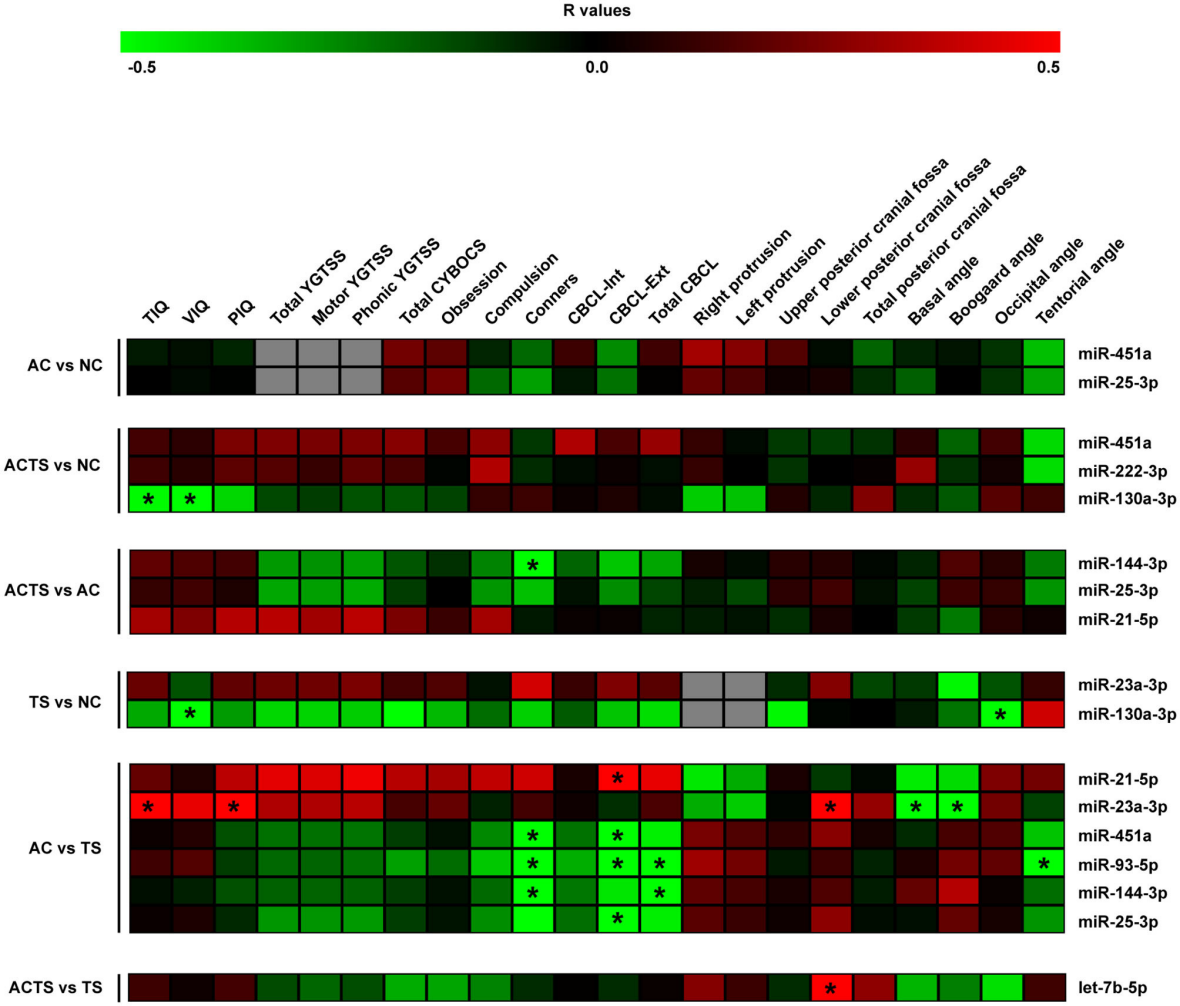
Correlation analysis of serum miRNA expression, cognitive impairments and neuroimaging measurements. Heat-maps of the correlations obtained by calculating Spearman correlation coefficients for miRNA expression, cognitive impairments, and neuroimaging measurements of the assessed participants and for each group of comparison. The correlation coefficient is indicated by a color gradient from green (negative correlation) to red (positive correlation), as shown in the colored bar. Statistically significant *p*-values (*p* < 0.05) are indicated by asterisks. TIQ, Total Intelligence Quotient; VIQ, Verbal Intelligence Quotient; PIQ, performance Intelligence Quotient; YGTSS, Yale Global Tic Severity Scale; Total CYBOCS, total Children Yale Brown Obsessive Compulsive Scale; CBCL-Int, Child Behavior Check list internalizing; CBCL-Ext, Child Behavior Check list externalizing; Total CBCL, total Child Behavior Check list.

Using a Spearman correlation analysis and applying two-sided *p*-values, the normalized counts of serum miRNAs were correlated with neuropsychological and neurological scores for each comparison (Figure 2).

By comparing ACTS and NC groups, we found a negative relationship between intelligent quotient scores (e.g., TIQ and VIQ) and miR-130a-3p (*r* = −0.49, *r* = −0.53, respectively), as well as between VIQ and miR-130a-3p (*r* = −0.65) by comparing TS and NC groups and a positive correlation between TIQ, PIQ and miR-23a-3p (*r* = 0.57, *r* = 0.54, respectively), in AC vs. TS comparison. We also found a negative relationship between Conners' scale and (i) miR-144-3p (*r* = −0.5) by comparing ACTS and AC groups and (ii) miR-451a (*r* = −0.8), miR-93-5p (*r* = −0.79), miR-144-3p (*r* = −0.79) comparing AC and TS groups. CBCL-ext scores were found positively correlated with miR-21-5p levels (*r* = 0.51) and negatively correlated with miR-451a (*r* = −0.52), miR-93-5p (*r* = −0.66), miR-25-3p (*r* = −0.50) in AC vs. TS comparison. From the same comparison, total CBCL scores were found negatively correlated with miR-93-5p (*r* = −0.67) and miR-144-3p (*r* = −0.50). On the other hand, neuroimaging measurements (e.g., lower posterior cranial fossa) were found positively correlated with miR-23a-3p (*r* = 0.63) by comparing AC and TS groups and let-7b-p (*r* = 0.53) by comparing ACTS vs. TS groups. From the same comparison, we found a negative correlation between Basal and Boogard angle measurements and miR-23a-3p (*r* = −0.66, *r* = −0.64, respectively). Finally, we found negative correlations between occipital angle measurements and miR-130a-3p (*r* = −0.56) by comparing TS vs. NC groups; tentorial angle and miR-93-5p (*r* = −0.63) by comparing AC vs. TS groups.

### Functional Enrichment Analyses

**Figure 3:**
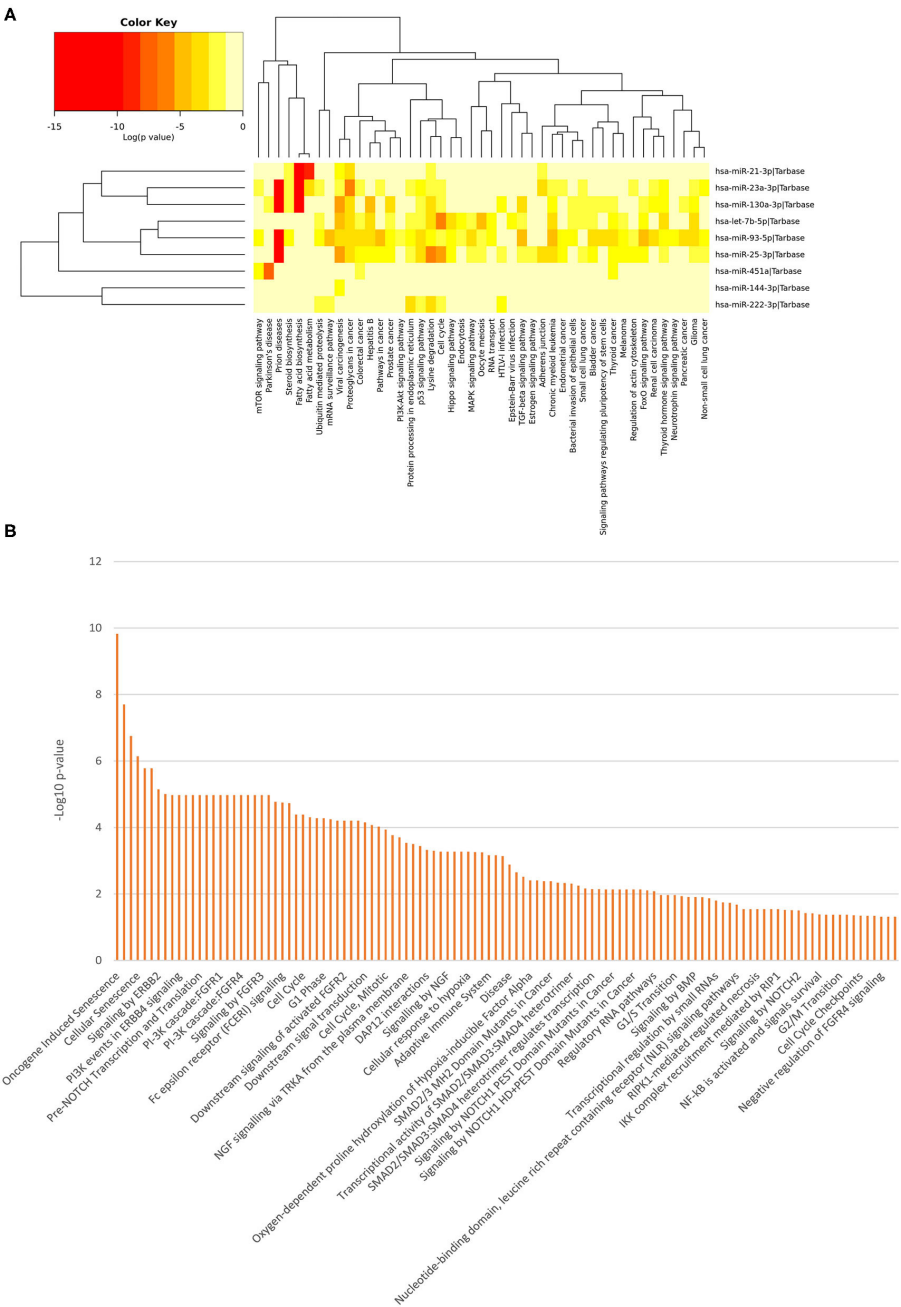
Functional Enrichment analysis of DE miRNAs. Functional enrichment analysis of all DE miRNA targets using KEGG pathway (hierarchical clustering based on a complete linkage method and the significance levels of the interactions) **(A)** and Reactome databases **(B)** by DIANA-mirPath v.3 web server and the miRNet tool, respectively.

In order to evaluate the potential biological impact related to the differential expression of all the miRNAs identified in this study, we computed pathway enrichment analyses. This analysis showed a list of statistically over-represented biological pathways (Figure 3), potentially related to the analyzed clinical conditions. Supplementary Figures 1–3 report all the data from the class-specific functional enrichment analysis, including those miRNAs found DE for each pathological condition with respect to the NC group.

Among the most interesting terms, we found signaling by NOTCH (*p* = 0.00000166), pre-NOTCH expression and processing signaling pathway (*p* = 0.0000106), NGF signaling via TRKA from the plasma membrane (*p* = 0.000293), signaling by NGF (*p* = 0.00053), NOTCH1 intracellular domain regulated transcription (*p* = 0.000551), signaling by NOTCH1 (*p* = 0.00391), constitutive signaling by NOTCH1 HD+PEST Domain Mutants (*p* = 0.00729), signaling by NOTCH2 (*p* = 0.0315), and signaling by Wnt (*p* = 0.0108), through which neuronal function and development are regulated. Moreover, we found pathways that mediate fear conditioning and behavior signaling by ERBB2 (*p* = 0.00000711) and ERBB4 (*p* = 0.0000106), also involved in the development of neurodegenerative diseases. Additionally, the PI3K/AKT activation pathway (*p* = 0.0000169) was also pinpointed, which is a major regulator of neuron survival, as well as FGFR1/2/3/4 (*p* = 0.0000106), signaling by FGFR1/2/3/4 (*p* = 0.0000106), downstream signaling of activated FGFR1/2/3/4 (*p* = 0.0000624), FoxO signaling pathway (*p* = 3.22E-07), Hippo signaling pathway (*p* = 1.58E-05), neurotrophin signaling pathway (*p* = 0.04106071) and recycling pathway of L1 (*p* = 0.0187), involved in neural development, including axon outgrowth and neuronal migration.

Other molecular signaling pathways were revealed, such as signaling by BMP (*p* = 0.0123), Ca2^+^ pathway (*p* = 0.0438), TGF-beta receptor signaling activates SMADs (*p* = 0.00053) and transcriptional activity of SMAD2/SMAD3:SMAD4 heterotrimer (*p* = 0.00491). These pathways are intriguing since they are related to disorders in bone tissue, muscle contraction, embryonic skeletal development, postnatal bone homeostasis dorsoventral polarity, which analyzed together suggest that the differential expression of these miRNAs could cause the developmental impairments leading to AC pathogenesis.

## Discussion

Chiari Type I malformation and Tourette Syndrome are heterogeneous disorders with a high degree of clinical variability and an elusive pathophysiology (Markunas et al., 2014; Cavanna, 2018; Singer and Augustine, 2019).

The complexity of phenotypes and their diagnostic recognition increases in comorbid conditions with other psychopathologies: it is unclear whether or not they all contribute to the genetic expression, or whether some etiological pathways components may be shared, as well as how the comorbid disorders influence each other (Robertson, 2000). To date, a coexistence of both AC and TS in the same individual (i.e., ACTS) has not been described. For this reason, in our study we designed to assess patients affected by AC, TS, NCs and comorbid ACTS, through standardized approaches for clinical diagnostic examinations (i.e., MRI and DSM-5, APA 2013).

Moreover, delineating neuropsychiatric disorders like TS is still challenging and needs potential molecular biomarkers, considering the etiological and clinical heterogeneity, the lack of an unbiased diagnostic test and no existing associations with any neuroimaging abnormality (Robertson, 2000; Cavanna, 2018).

Therefore, we hypothesized that serum profiles of circulating miRNAs may contain molecular fingerprints for AC, TS, and ACTS, which could support the clinical discrimination process.

MiRNAs play pivotal roles in the regulation of complex networks and biological pathways. Their deregulated expression is associated with disease pathogenesis, including neuropsychiatric disorders (Alural et al., 2017). Analyzing miRNA expression profiles in body fluids could reflect the real pathophysiologic processes of patients through very simple and non-invasive approaches, since brain tissue is not easily accessible (Kichukova et al., 2015).

Despite many studies investigating whether miRNA might contain specific fingerprints for neurological and psychiatric disorders (Fineberg et al., 2009; Xu et al., 2012; De Sena Cortabitarte et al., 2018; Nguyen et al., 2018; Tonacci et al., 2019), only a few included Tourette syndrome (Rizzo et al., 2015; Cirnigliaro et al., 2017) and none is focused on AC and ACTS.

Through a high-throughput approach, we investigated cmiRNA expression in the sera of 10 AC, 11 ACTS, 6 TS, and 8 unaffected (NC) subjects. Results showed the identification of nine serum miRNAs as significantly differentially expressed in different groups of comparison (Figure 1). Interestingly, we observed that DE miRNAs in comparisons among ACTS, AC, and TS groups (i.e., miR-451a, miR-21, miR-25-3p, miR-144-3p, 5p) showed a marked different expression trend between AC and ACTS groups, unlike that between ACTS and TS (Figure 1). This observation suggests that miRNA expression could be mostly affected by the typical TS impairments and physiological conditions, when in comorbid conditions. Further investigations are needed to evaluate this hypothesis.

The nine DE miRNAs identified in this study, had been previously identified as dysregulated in cellular and extra-cellular frameworks of other neurological and/or bone disorders, suggesting a critical role in developing cognitive functions and bone morphogenesis (Gao et al., 2011; Mor et al., 2015; Xu et al., 2015; Hicks and Middleton, 2016; Hicks et al., 2016; Yan et al., 2016; Cirnigliaro et al., 2017; Fujiwara et al., 2017; Zhang et al., 2017; Yang et al., 2019).

Molecular pathways identified in our functional enrichment analysis would support the involvement of these miRNAs in AC and TS mechanisms. Among these, the Wnt pathway could be associated with AC pathogenesis by affecting the bone homeostasis and bone remodeling process (Monroe et al., 2012). Wnt pathway is also involved in a crosstalk with Bone Morphogenetic Protein (BMP) and TGF-β signaling during osteoblast and chondrocyte differentiation (Wu et al., 2016). As key regulators of TGFβ-induced chondrogenesis of human mesenchymal stem cells (BMSCs), activated SMAD2 and SMAD3 proteins form complexes with co-factor SMAD4 to regulate gene transcription (Heldin et al., 1997; De Kroon et al., 2017).

Notch signaling, in bone marrow, plays an important role determining the maintenance of a pool of mesenchymal progenitors by suppressing osteoblast differentiation (Hilton et al., 2008). Furthermore, alterations in signaling by Notch and NGF could have important effects on neurogenesis and neurodevelopmental processes (Chao et al., 2006; Lathia et al., 2008), together with Hippo signaling (Wang and Wang, 2016), PI3K/AKT activation (Cuesto et al., 2011), FoxO signaling (Polter et al., 2009) and signaling by ERBB (Mei and Nave, 2014), which overall control neuronal maintenance, as well as behavioral manifestation, and may be crucial for TS pathogenesis.

Finally, we evaluated whether a relationship could exist between miRNA expression and TS and AC clinical parameters, commonly used in the diagnostic evaluation.

We found several linear correlations between miRNA serum expression levels, intelligent quotient scores, cranial disproportions, and behavioral impairments (Figure 2).

Among the most interesting, the lower concentrations of miR-144-3p in ACTS group, and miR-451a,−93-5p,−144-3p in TS, both compared to AC group, have been found associated to higher Conners' scale scores, commonly used to measure behavioral problems. The associations between lower concentrations of miR-144-3p and higher Conner's scale scores both in TS and ACTS vs. AC, suggest that the TS phenotype, in comorbid condition, could contribute to impair serum abundance of this miRNA more strongly than AC phenotype. This hypothesis is consistent with the markedly different expression of miR-144-3p between the AC and ACTS groups, differently than ACTS and TS comparison showing no difference (Figure 1). Moreover, we found significant correlations between the abundance of miR-21-5p,−451a,−93-5p,−144-3p,−25-3p in TS, compared to AC group, and higher CBCL scores, which assessed behavior impairments in children.

Overall, the multiplicity of such correlations strongly suggests that the miRNA differential abundance in serum is potentially associated with the occurrence and worsening of behavioral impairments related to TS psychopathology.

Other interesting associations between some DE miRNAs and neuroimaging measurements were revealed. Among these, by comparing the AC vs. TS groups, we found a relevant association between the lower serum concentrations of miR-23a-3p and the smaller area measures of the lower PCF in AC group, which is a clinical hallmark of AC patients (Marin-Padilla and Marin-Padilla, 1981; Milhorat et al., 2010).

Taken together, these associations might indicate that miRNA profiling are consistent with clinical findings and could potentially support the current diagnostic evaluation.

Despite the exploratory nature and the limited sample size, due to the rarity of the pathological conditions examined, our study offers new insights into the molecular bases of AC, TS, and ACTS diseases. The preliminary results strongly encourage further investigations in independent cohorts that could validate the diagnostic power of the identified circulating miRNAs as appropriate non-invasive biomarkers.

## Data Availability Statement

The datasets generated for this study can be found in online repositories. The names of the repository/repositories and accession number(s) can be found in the article/Supplementary Material.

## Ethics Statement

The studies involving human participants were reviewed and approved by Comitato Etico Catania 1. Written informed consent to participate in this study was provided by the participants' legal guardian/next of kin.

## Author Contributions

RR, MR, and MP conceived the project and planned the experiments. MG and MCa carried out patient recruitment. RR performed the clinical diagnosis. SP performed magnetic resonance scans and neuroimaging measurements. FM, GL, and MCi performed the experiments. FM, GL, MCi, DB, and MS performed the bioinformatics analysis and data statistics. MR and FM wrote the manuscript. RR, MR, MP, and CDP revised the manuscript. All authors read and approved the final manuscript.

## Conflict of Interest

The authors declare that the research was conducted in the absence of any commercial or financial relationships that could be construed as a potential conflict of interest.
