# Supplementary material for: Enrichment and Correlation Analysis of Serum miRNAs in Comorbidity Between Arnold-Chiari and Tourette Syndrome Contribute to Clarify Their Molecular Bases
Source: Front Mol Neurosci. 2021 Jan 5;13:608355. doi: 10.3389/fnmol.2020.608355 (PMC7813987; doi:10.3389/fnmol.2020.608355)
Supplement: Supplementary file 2 [file Data_Sheet_2.PDF]

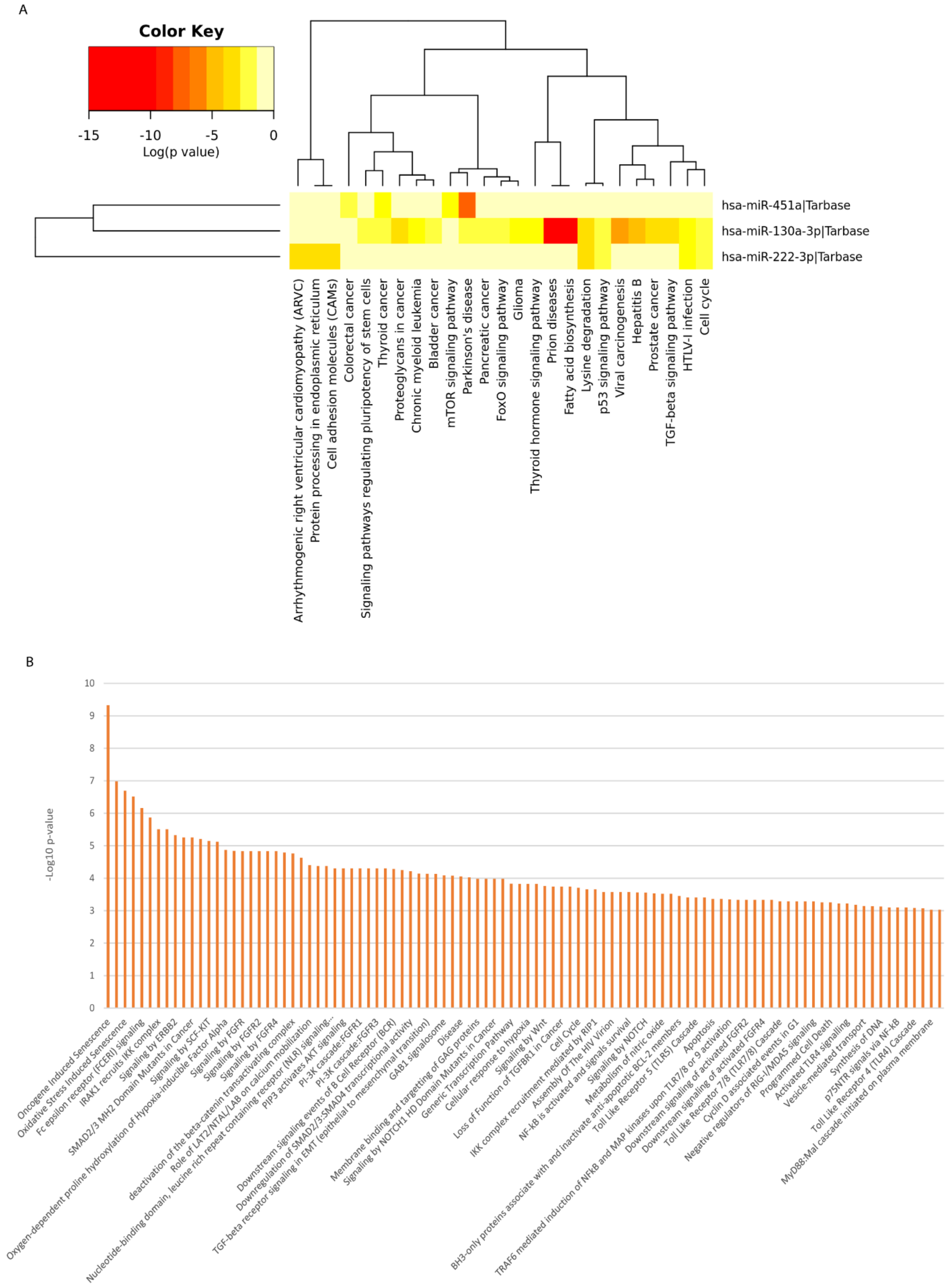

Supplementary Figure 2. Functional Enrichment analysis of DE miRNAs in comparison among ACTS and NC groups.

Functional enrichment analysis of DE miRNA targets using KEGG pathway (hierarchical clustering based on a complete linkage method and the significance levels of the interactions) (A) and Reactome databases (B) by DIANA-mirPath v.3 web server and the miRNet tool, respectively.
